# Differential Host Immune Responses after Infection with Wild-Type or Lab-Attenuated Rabies Viruses in Dogs

**DOI:** 10.1371/journal.pntd.0004023

**Published:** 2015-08-20

**Authors:** Clement W. Gnanadurai, Yang Yang, Ying Huang, Zhenguang Li, Christina M. Leyson, Tanya L. Cooper, Simon R. Platt, Stephen B. Harvey, Douglas C. Hooper, Milosz Faber, Zhen F. Fu

**Affiliations:** 1 Department of Pathology, College of Veterinary Medicine, University of Georgia, Athens, Georgia, United States of America; 2 State-key Laboratory of Agricultural Microbiology, College of Veterinary Medicine, Huazhong Agricultural University, Wuhan, China; 3 Department of Population Health, College of Veterinary Medicine, University of Georgia, Athens, Georgia, United States of America; 4 Small Medicine & Surgery, College of Veterinary Medicine, University of Georgia, Athens, Georgia, United States of America; 5 Department of Cancer Biology and Neurological Surgery, Thomas Jefferson University, Philadelphia, Pennsylvania, United States of America; The Global Alliance for Rabies Control, UNITED STATES

## Abstract

Rabies virus (RABV) induces encephalomyelitis in humans and animals. One of the major problems with rabies is that the infected individuals most often do not develop virus neutralizing antibodies (VNA). In this study we have investigated the host immune response to RABV infection in dogs, using a live-attenuated (TriGAS) or a wild-type (wt) (DRV-NG11) RABV isolated from a rabid dog.

**Methodology/Principal Findings:**

The experimental infection of dogs with TriGAS induced high levels of VNA in the serum, whereas wt RABV infection did not. Dogs infected with TriGAS developed antibodies against the virus including its glycoprotein, whereas dogs infected with DRV-NG11 only developed rabies antibodies that are presumably specific for the nucleoprotein, (N) and not the glycoprotein (G). We show that infection with TriGAS induces early activation of B cells in the draining lymph nodes and persistent activation of DCs and B cells in the blood. On the other hand, infection with DRV-NG11 fails to induce the activation of DCs and B cells and further reduces CD4 T cell production. Further, we show that intrathecal (IT) immunization of TriGAS not only induced high levels of VNA in the serum but also in the CSF while intramuscular (IM) immunization of TriGAS induced VNA only in the serum. In addition, high levels of total protein and WBC were detected in the CSF of IT immunized dogs, indicating the transient enhancement of blood-brain barrier (BBB) permeability, which is relevant to the passage of immune effectors from periphery into the CNS.

**Conclusions/Significance:**

IM infection of dogs with TriGAS induced the production of serum VNA whereas, IT immunization of TriGAS in dogs induces high levels of VNA in the periphery as well as in the CSF and transiently enhances BBB permeability. In contrast, infection with wt DRV-NG11 resulted in the production of RABV-reactive antibodies but VNA and antibodies specific for G were absent. As a consequence, all of the dogs infected with wt DRV-NG11 succumbed to rabies. Thus the failure to activate protective immunity is one of the important features of RABV pathogenesis in dogs.

## Author Summary

Remarkable advances have been made in elucidating the pathogenesis of rabies. One of the hallmarks of rabies infection is that human patients do not develop VNA. In this present study, immune responses were investigated in dogs after infection with a highly attenuated RABV (TriGAS) or a highly pathogenic, truly wt RABV (DRV-NG11). DRV-NG11 was isolated from the brain of a rabid dog and has not been passaged in laboratory animals or in cell culture. IM infection of dogs with TriGAS induced early activation of DCs and B cells in the blood, and production of VNA in the periphery, whereas, IT infection with TriGAS induced a high level of VNA not only in the serum but also in the CSF. On the contrary, infection of dogs with DRV-NG11 failed to elicit VNA. As a result, none of the dogs infected with wt DRV-NG11 survived. The ability of DRV-NG11 to replicate and spread without triggering an immune response to glycoprotein is evidently an important facet of its pathogenicity.

## Introduction

Rabies is a neurological disease in humans and other warm-blooded animals caused by rabies virus (RABV). It is transmitted by animal bites or scratches. Despite the advances in the development of rabies biologicals, rabies remains a public health threat, causing more than 55,000 human deaths every year around the globe [1]. A hallmark of wild-type (wt) RABV is its unique ability to invade the central nervous system (CNS) from the peripheral inoculation/bite sites through the neuromuscular junction via motor or sensory neurons [2, 3].

Once the RABV enters the nervous system, it successfully evades the host immune responses [4, 5]. One of the mechanisms that contribute to rabies immune evasion and pathogenesis, is the low level of viral replication and subsequent preservation of neuronal structures by minimal viral antigen exposure to the host immune system [6]. It has been known for a long time that most of the human rabies patients (>70%) do not develop virus neutralizing antibodies (VNA) prior to or at the time of death [7]. It has also been reported that wt RABV infection is unable to induce VNA responses in other animal species such as mice [8], dogs and skunks [9, 10]. On the other hand, attenuated RABV replicates rapidly, expresses large amount of the glycoprotein (G), induces strong immune responses in laboratory animals and protects the animals from subsequent lethal challenge [11–13]. Although the mechanism(s) by which different RABV induce differential immune responses are unknown, recent studies indicate that laboratory-attenuated RABV activates, while wt RABV evades, the host innate immune responses [8]. However, most of the studies to evaluate the differential host immune responses to laboratory-attenuated or wt RABV infection were carried out in small laboratory animals such as mice, rabbits and rats, which are not the natural host for rabies. Furthermore, most of the wt or pathogenic RABVs used in these studies were at least passaged or amplified in suckling mouse brain, which might alter their phenotypes [14, 15].

Although remarkable progress has been made in the understanding of rabies pathogenesis in laboratory animals or *in vitro* [16, 17], rabies remains almost always fatal once clinical signs appear. The only available treatment for rabies is the “Milwaukee Protocol”, however because of recent failures, practicing the “Milwaukee Protocol” is not considered as an appropriate step towards establishing an effective treatment for rabies [18–21]. It is crucial to understand the basic mechanism(s) of rabies pathogenesis in order to develop effective treatment for rabies. The six non-lethal human rabies cases that have been documented in the USA indicate that all these patients had rabies-specific VNA in the CSF at the time of hospitalization or after treatment [22–24]. Presence of VNA in the CSF has also been observed in laboratory animals such as mice [25], ferrets [26], and dogs [27, 28], which correlates with non-lethal rabies infection or recovery. These observations highlight the importance of VNA in the CSF and the clearance of RABV from the CNS [27]. It has been shown most recently that it is possible to clear an established infection with wt RABV by intravenous administration of VNA in both immunocompromised and immunocompetent mice as long as the BBB permeability is enhanced [29]. It has also been shown in rabid rabbits that it is possible to reverse the course of infection by intrathecal immunization with attenuated RABV [30].

In the present study, induction of immune responses was investigated in dogs after infection with a highly attenuated TriGAS or a highly pathogenic and a truly wt DRV-NG11. It was found that the TriGAS infection induced high levels of VNA and anti-RABV (binding antibodies) in the serum, whereas wt RABV induced only binding antibodies but not VNA.. Analysis of anti-RABV antibodies indicates that both attenuated and wt RABVs induced comparable levels of anti-RABV antibodies. However, the anti-RABV G antibodies were significantly higher in dogs infected with TriGAS than in those infected with DRV-NG11 or uninfected dogs. TriGAS infection induced activation of immune cells, particularly DCs and B cells, whereas infection with DRV-NG11 failed to induce activation of immune cells. As a consequence, all the dogs infected with wt DRV-NG11 succumbed to rabies. However, the dogs infected IM with TriGAS resisted a subsequent lethal challenge with wt RABV. Together, these data demonstrate that lab-attenuated RABV activates, while pathogenic RABV fails to induce the production of VNA, the most important component for rabies protection.

## Methods

### Viruses and animals

A canine RABV, DRV-NG11, was originally isolated from a dog in Nigeria [31]. Virus stocks were prepared by inoculating 100μl of the brain suspension into another dog. The dog was euthanized and its brain removed after showing typical signs of rabies. A 10% (w/v) suspension was prepared by homogenizing the brain in DMEM. The homogenate was centrifuged to remove debris and the supernatant collected and stored at -80°C. TriGAS has incorporated the known change (mutation of amino acid residue at position 333 from Arg to Glu) that attenuates RABV [32]. The recombinant RABV is further attenuated by expressing three copies of the G [12]. In addition, Asn194→Ser194 mutation stabilizes the attenuated phenotype [33].

### Dog vaccination and infection

Dogs were infected IM with either TriGAS or DRV-NG11 by direct inoculation into the left hemisphere of the temporalis muscle. Dogs immunized with TriGAS were challenged 4 weeks post immunization with DRV-NG11. Challenged dogs were observed at least once a day prior to challenge and three times a day for 90 days after challenge. Humane endpoint of the study was considered to be the appearance of hind limb paralysis of one or both limbs and the experimental endpoint of the study was determined on the basis of observed clinical signs. Blood, lymph node aspirate, CSF and brain samples were collected before infection and/or at the time of termination for various analyses.

For IT vaccination, dogs were pretreated with midazolam (0.06–0.3 mg/kg) and ketamine (6.6–11 mg/kg). When unconscious, the dogs were transported to the procedure room and anesthetized with propofol (3–4 mg/kg IV) for endotracheal intubation and maintained with isoflurane (2–4%) during the IT injection. Initially, 1 ml of CSF was collected and then a single dose of 10^7^ FFU of attenuated TriGAS was injected into the IT space. Mild hyper immune reactions were seen in animals and were then treated accordingly.

### Clinical pathology

The CSF samples were collected before and after infection and sent to the Clinical Pathology Laboratory at University of Georgia for analysis. The CSF samples were collected from the cerebello- medullary cistern and were evaluated for white blood cell counts (WBC) and total protein concentration. The CSF protein was measured on the Roche Hitachi P module.

### Flow cytometry

The blood and lymph node aspirates were collected before and after infection. Briefly, PBMCs were isolated from 10ml of blood using Ficoll Histopaque solution (Sigma-Aldrich). Then, cells were stained with CD3, CD4, CD8, CD19, CD40, CD11c and CD86 antibodies and isotype control. Data collection and analysis were performed using a BD LSR-II flow cytometer, BD FACSDiva software (BD Pharmingen), and FlowJo software (TreeStar, San Carlos, CA).

### Evaluation of antibody responses to rabies infection by ELISA

Sera obtained from dogs were tested to analyze the humoral responses to wt-RABV or TriGAS infection by ELISA. In order to determine the antibody responses against rabies infection, dog rabies virus and dog anti-rabies virus glycoprotein IgG ELISA kits from “Alpha Diagnostic” were used. Briefly, 100μl of diluted standards, controls and sera obtained from dogs were pipetted into the respective antigen coated 96 well plates and incubated at room temperature for 1 hour. Plates were then washed four times with wash buffer and 100μl of conjugate containing anti-dog IgG HRP was added. After 30 minutes incubation, plates were washed five times and 100μl of TMB substrate was added and incubated for 15 minutes. The color development was stopped by stop solution and plates were measured for OD at 450nm, using “BioTek” ELISA plate reader.

### RFFIT

Blood and CSF samples were collected for measurement of VNA using the RFFIT (Rapid Fluorescent Focus Inhibition Test) as described previously [34]. Briefly, 50 μl of serial three-fold dilutions of serum were prepared in 96 well plates (Nalge Nunc International, Rochester, NY). Fifty FFD50 (50%Focus Forming Dose) of CVS-11 was added to each well and incubated for 90 min at 37°C. NA cells (10^5^ cells) were added into each well and the plates were incubated in a CO_2_ controlled incubator at 37°C for 20 hr, fixed with ice-cold 80% acetone and stained with FITC-conjugated anti-RABV N antibodies (Fujirebio Diagnostics, Inc.) for 1 hr at 37°C. Twenty fields in each well were observed under a fluorescent microscope using 10X objective, and the 50% endpoint titers were calculated according to the Reed-Muench formula [35]. The values were compared with that of reference serum (obtained from the National Institute for Biological Standards and Control, Herts, UK) and normalized to international units (IU/ml). Lower limit of detection is 0.1 IU/ml [36].

### Ethics statement

The project AUP is entitled, ‘‘Virus clearance from the central nervous system” and the AUP number is A2011 03–016. It was approved by the University of Georgia’s Institutional Animal Care and Use Committee on 4^th^ APR 2011. The University of Georgia’s University Research Animal Resources unit is fully accredited by the Association for Assessment and Accreditation of Laboratory Animal Care, International (AAALAC-I). The registration number from the U.S. Department of Agriculture, Animal and Plant Health Inspection Service, Animal Care is (USDA APHIS-AC). We have an assurance on file with the NIH-Office of Laboratory Animal Welfare (NIH-OLAW), and are in compliance with the PHS Policy on Humane Care and Use of Laboratory Animals and the 8th edition of the Guide for the Care and Use of Laboratory Animals, 2011.

### Statistical analysis

Statistical significance of the differences between groups was tested using student’s T test with *** indicating a p value< 0.0001, ** a p value< 0.001, and * a p value< 0.05 using Graph Pad prism software.

## Results

### 1) IM immunization with a single dose of attenuated TriGAS confers protective immunity against rabies in dogs

**Fig 1:**
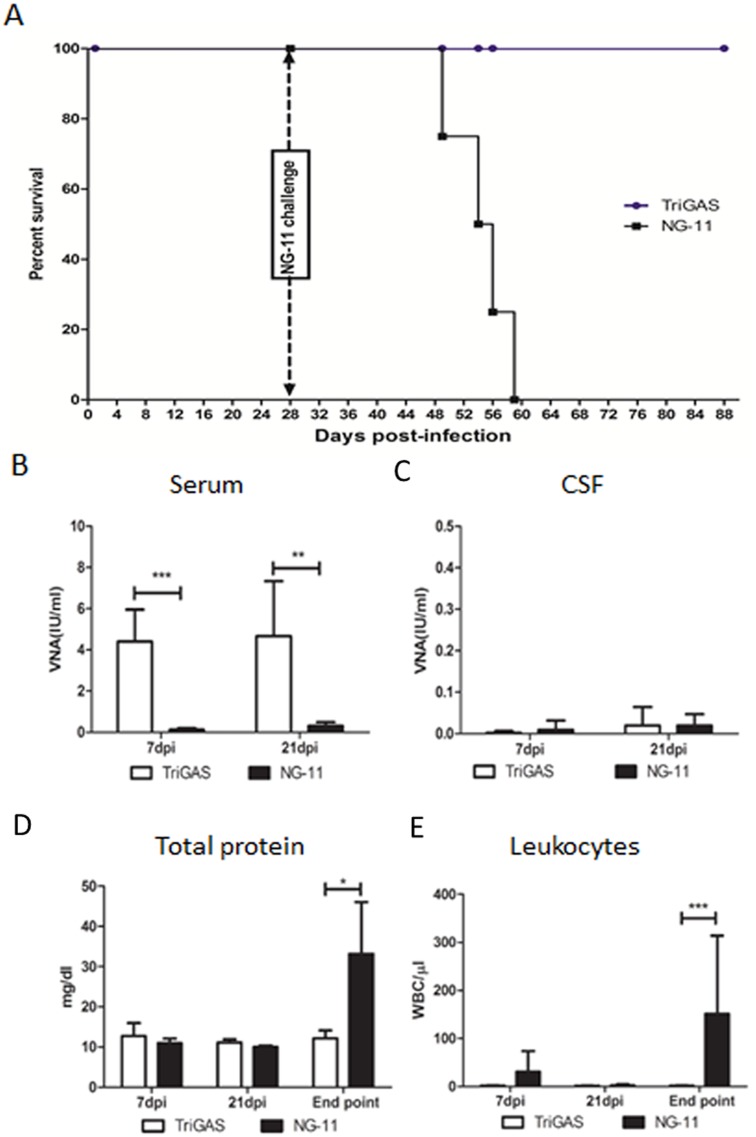
(A) Group of dogs were IM infected with a single dose of 10^7^ FFU of live-attenuated TriGAS. At 4 weeks after immunization dogs were challenged IM with 200 MICLD_50_ of DRV-NG11. As control, group of unvaccinated dogs were infected IM with 200 MICLD_50_ of DRV-NG11, and their survivorship were recorded for 60 days. (B) VNA in the serum. (C) VNA in the CSF. (D) Analysis of CSF total proteins. (E) Leukocytes in the CSF.

In order to investigate the immune responses in dogs after infection with attenuated or wt RABV, a group of five dogs were IM infected with a single dose of 10^7^ FFU of attenuated TriGAS and observed for 28 days. None of these dogs developed any clinical signs during the observation period. Four weeks after infection, the dogs were challenged with 300μl of viral suspension containing 200 MICLD_50_ (median mouse intracerebral lethal dose) of DRV-NG11 by direct inoculation into the right hemisphere of the temporalis muscle. Again none of these dogs developed any clinical signs after challenge with wt RABV. Another group of 4 dogs were infected IM only with 300μl of viral suspension containing 200 MICLD_50_ of DRV-NG11 by direct inoculation into the left hemisphere of the temporalis muscle. All the dogs were observed three times a day for another 3 months for any rabies related clinical signs. All of these dogs developed clinical signs at 19, 25, 26 and 30 days post infection which included behavior changes (quietness, lethargy and loss of appetite), high-pitched barking, poor coordination, agitation, trembling, persistent regurgitation and retching, excess salivation and lameness or paralysis. Hind limb paralysis of one or both limbs was used as the final humane end point. All the animals reached the humane end point and were sacrificed on 21, 26, 28 and 31 dpi, respectively (Fig 1A). Thus, IM immunization with a single dose of attenuated TriGAS confers protective immunity against rabies in dogs.

### 2) IM infection with a single dose of attenuated TriGAS but not wt DRV-NG11 induced high level of serum VNA in dogs

To determine the immune responses after infection, serum and CSF samples collected at various time points were subjected to RFFIT for VNA analysis and the results were expressed as mean values of the group. As shown in Fig 1B, dogs infected with TriGAS developed serum VNA titers of 4.4 and 4.6 IU/ml at 7 and 21 days, respectively. The VNA titers in these dogs increased to 5.8 and 18.3 IU/ml at 7 and 21 days post challenge, respectively. However, dogs infected with DRV-NG11 had only 0.12 and 0.32 IU/ml of serum VNA titers at 7 and 21 dpi, respectively, far below the 0.5 IU/ml, a level considered by WHO as a correlate for adequate vaccination or protection [1]. No VNA was detected in the CSF of dogs infected with either virus (Fig 1C). These results indicate that the attenuated TriGAS infection induces high level of VNA in serum, whereas wt DRV-NG11 induces little to no VNA responses in dogs.

### 3) Wt DRV-NG11, but not attenuated TriGAS, induces severe influx of WBCs and total protein into the CSF during the terminal stage of infection

Next, we analyzed the CSF samples collected at various time points for the quantification of total protein and also for the presence of white blood cells (WBC), as an indicator for BBB integrity. As shown in Fig 1D, significantly higher levels of CSF total protein (33.1 mg/dl) were detected at the terminal stage in dogs infected with DRV-NG11 but not in dogs infected with the attenuated TriGAS (11.1 mg/dl) (Fig 1D). Also, analysis of WBC in the CSF indicates (Fig 1E) that significant influx of WBC into the CSF (151/μl) was found at the terminal stage in dogs infected with DRV-NG11 but not in dogs infected with the attenuated TriGAS (1/μl). Thus, these results indicate that infection with wt DRV-NG11, but not attenuated TriGAS, leads to BBB damage at the terminal stage of infection in dogs.

### 4) Both the TriGAS and DRV-NG11 induced higher levels of anti-RABV antibodies than the uninfected controls while only TriGAS induced anti-RABV G antibodies

**Fig 2:**
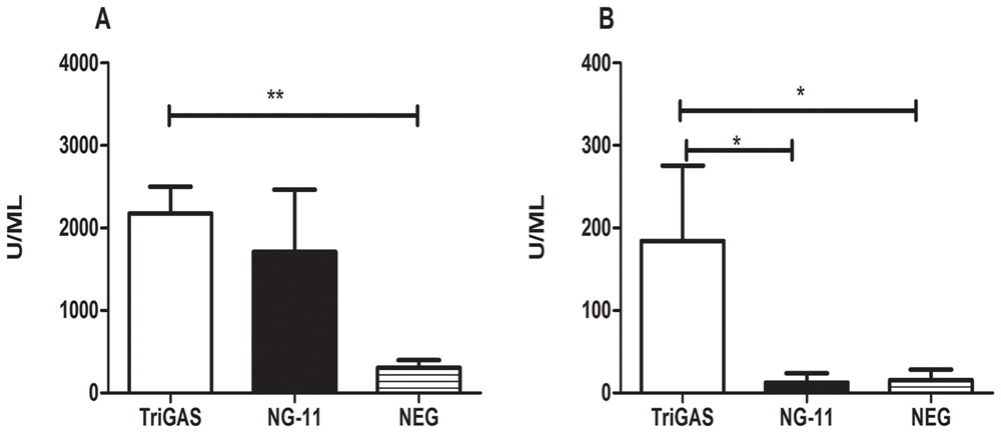
TriGAS induced both anti-RABV and anti-G but wt RABV induced anti-RABV, but not anti-G antibodies as detected by ELISA. A) Quantification of anti-RABV antibodies in the serum by ELISA. B) Quantification of anti-G antibodies in the serum by ELISA.

In order to assess whether the induction of VNA is RABV-specific or G-specific, serum samples were subjected to quantification of anti-RABV or anti-G antibodies by ELISA. As shown in Fig 2A, attenuated TriGAS induced higher levels of anti-RABV antibodies (2171 U/ml) than the uninfected controls (305 U/ml). Wt DRV-NG11 induced anti-RABV antibodies of 1952U/ml. Although it is higher than the uninfected controls (305 U/ml), but not significantly different from either the level induced by TriGAS or the uninfected controls. These results indicate that both attenuated and wt RABVs are capable of inducing similar level of antibodies against rabies infection. Next, we quantified the level of antibodies induced specifically against rabies G in the serum of dogs. As shown in Fig 2B, the anti-RABV G antibodies were 184 U/ml in dogs infected with TriGAS, whereas the anti-RABV G antibodies were only 12 & 15 U/ml, respectively in uninfected dogs and dogs infected with DRV-NG11 (Fig 2B). These results indicate that only the attenuated, but not the wt, RABV has the ability to induce G-specific antibodies.

### 5) Lab-attenuated RABV induces activation of immune cells in the LN and in the blood at the early stage of infection, while wt RABV fails to activate

**Fig 3:**
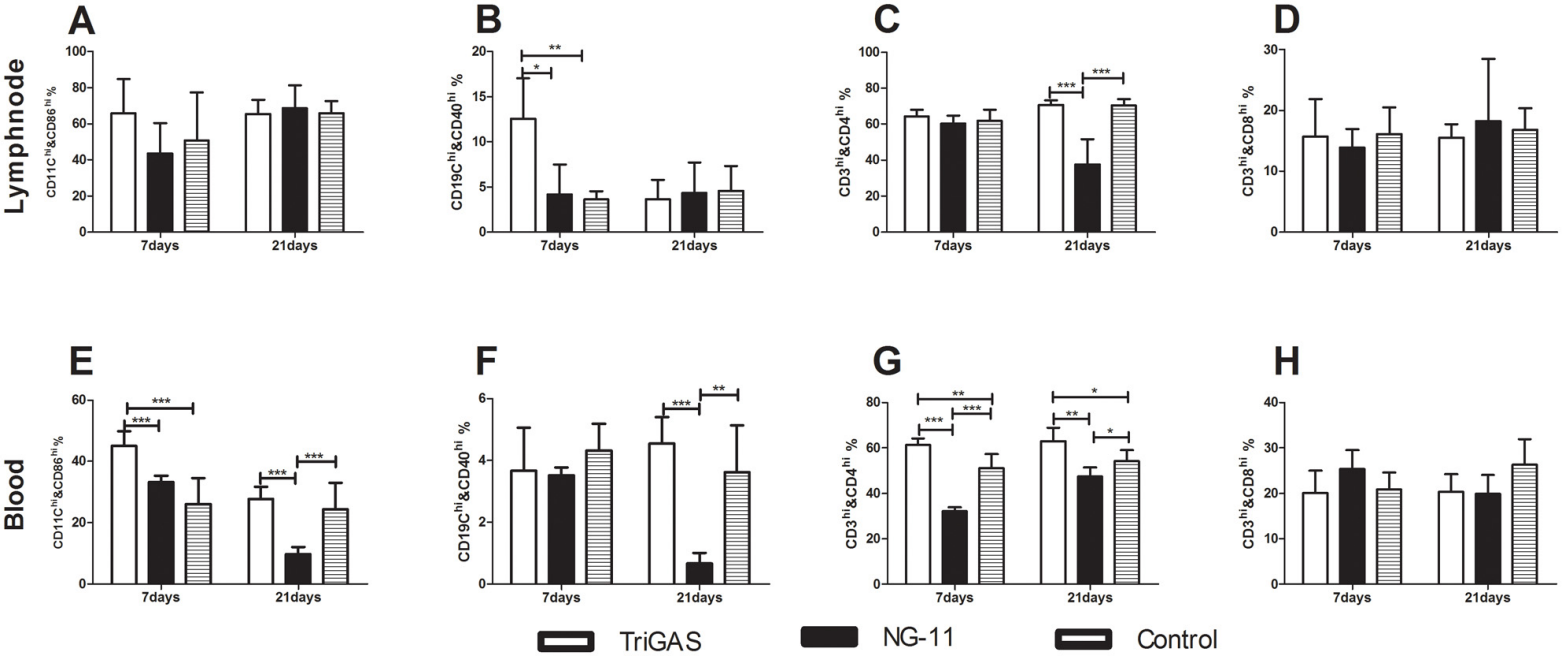
Lab-attenuated RABV induces early activation of immune cells while wt RABV evades immune activation. Groups of dog were IM injected with attenuated TriGAS or wt NG-11. Flow cytometric analysis of activation status of DCs and B-cells, and number of CD4 and CD8 T-cells in the lymph node (A-D) and in the blood (E-H).

In order to investigate the activation status of immune cells, blood and fine needle aspirate cytology (FNAC) samples of lymph node were collected at various time points after infection for flow cytometric analysis. As shown in Fig 3, significantly greater number of activated B cells and DCs, and CD4+ T cells were detected in the LN and the blood of dogs infected with attenuated than in those infected with wt RABV at 7 dpi. However, the number of CD4+ T cells in the blood was significantly lower in dogs infected with wt RABV at 7dpi (Fig 3G). By 21 dpi, significantly fewer number of DCs, B cells in the blood (Fig 3E and 3F) and CD4+ T cells in both the blood and the LN of dogs infected with wt than in those infected with attenuated RABV (Fig 3C and 3G). Together, these results demonstrate that attenuated TriGAS activates, while the wt RABV suppresses the activation of the immune cells.

### 6) IT immunization with a single dose of attenuated TriGAS induces high level of VNA in the serum and the CSF

It has been shown that the presence of rabies VNA in the CSF is critical for the clearance of RABV from the CNS or recovery from rabies [26, 27, 37]. In order to determine the immune responses induced after IT vaccination, dogs were vaccinated IT with attenuated TriGAS. Initially, 1 ml of CSF was collected and then a single dose of 10^7^ FFU of the attenuated TriGAS was injected into the intrathecal space. At 7 and 21 days post immunization, blood and CSF samples were collected for various analyses.

**Fig 4:**
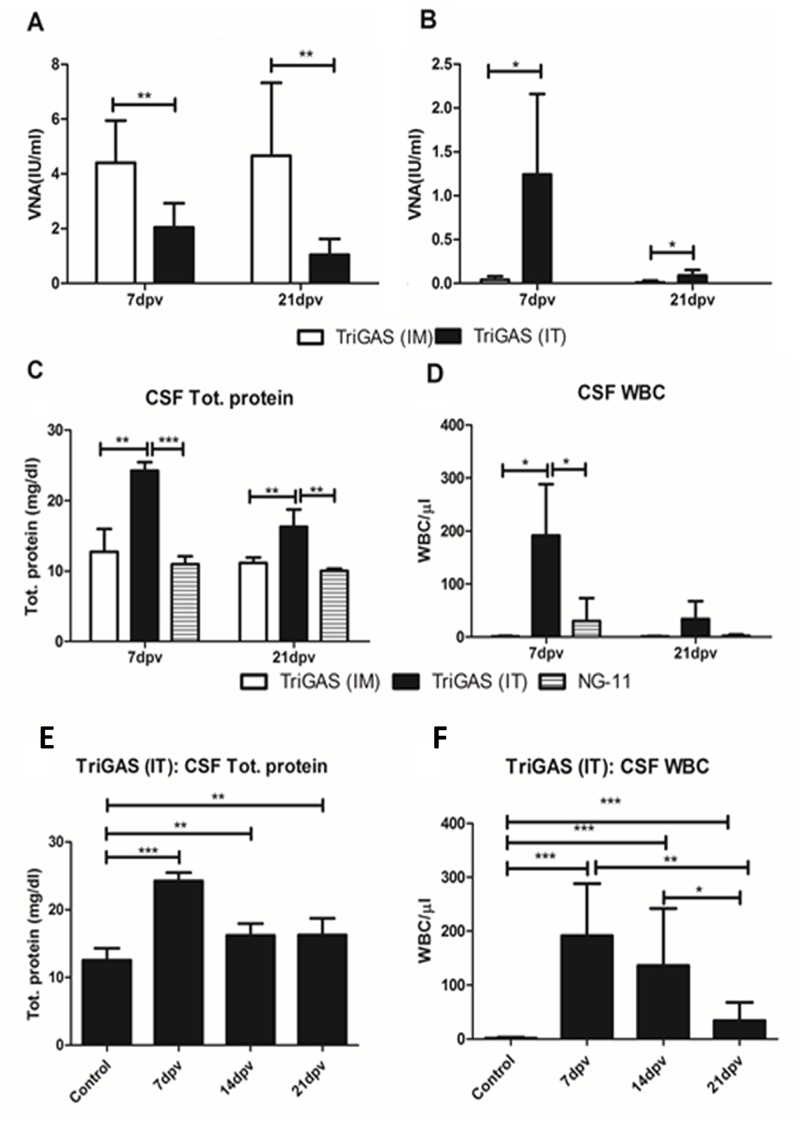
Intrathecal (IT) immunization with a single dose of laboratory-attenuated TriGAS induces high level of VNA in the serum and the CSF, and induces transient opening of blood-brain barrier in dogs. VNA detected in serum (A) and in CSF (B). Analysis of total protein (C) leukocytes (D) in the CSF. Kinetics of total protein (E) and leukocytes (F) influx into the CSF.

Serum and CSF samples collected at various time points were subjected to RFFIT for VNA analysis. As shown in Fig 4A, comparison of VNA between IM and IT vaccinated groups indicate that the IM vaccinated dogs had serum VNA titers of 4.4 and 4.6 IU/ml at 7 and 21 days post immunization, and the IT vaccinated dogs had a serum VNA of 2.2 and 1.2 IU/ml at 7 and 21 days post immunization, respectively (Fig 4A). However, analysis of CSF VNA indicate that, only the IT vaccinated dogs had VNA of 1.2 and 0.2 IU/ml at 7 and 21 days post immunization. No VNA was detected in the CSF of dogs that were infected IM with attenuated TriGAS (Fig 4B). These results indicate that the IT immunization with attenuated TriGAS can induce adequate level of VNA (>0.5IU/ml) both in the serum and in the CSF in dogs.

### 7) IT immunization with a single dose of attenuated TriGAS induces transient opening of BBB in dogs

Next, we compared the total protein and WBC levels in the CSF samples collected at day 7 and 21 post IT or IM immunization. As shown in Fig 4C, significantly higher level of total protein (24.3 and 16.2 mg/dl) were detected in the CSF of IT immunized dogs than the IM immunized dogs (12.7 and 11.4 mg/dl) at 7 and 21 days, respectively (Fig 4C). Analysis of CSF-total protein in IT immunized dogs at various time points indicates that the level of CSF-total protein peaked significantly at 7 days post immunization (Fig 4E). Similarly, significantly more number of CSF-WBCs was detected in IT immunized-dogs at 7 days post vaccination than in the IM immunized dogs (Fig 4D). Also, analysis of WBC in the CSF at different time points indicate that influx of WBC into the CSF peaked at day 7 and declined by 21 days post IT immunization (Fig 4F). Thus, these results indicate that the IT injection of attenuated TriGAS leads to transient opening of BBB, thereby higher levels of VNA, total protein and WBCs in the CSF.

### 8) Comparison of immune responses in dogs by IM and IT immunization with TriGAS

**Fig 5:**
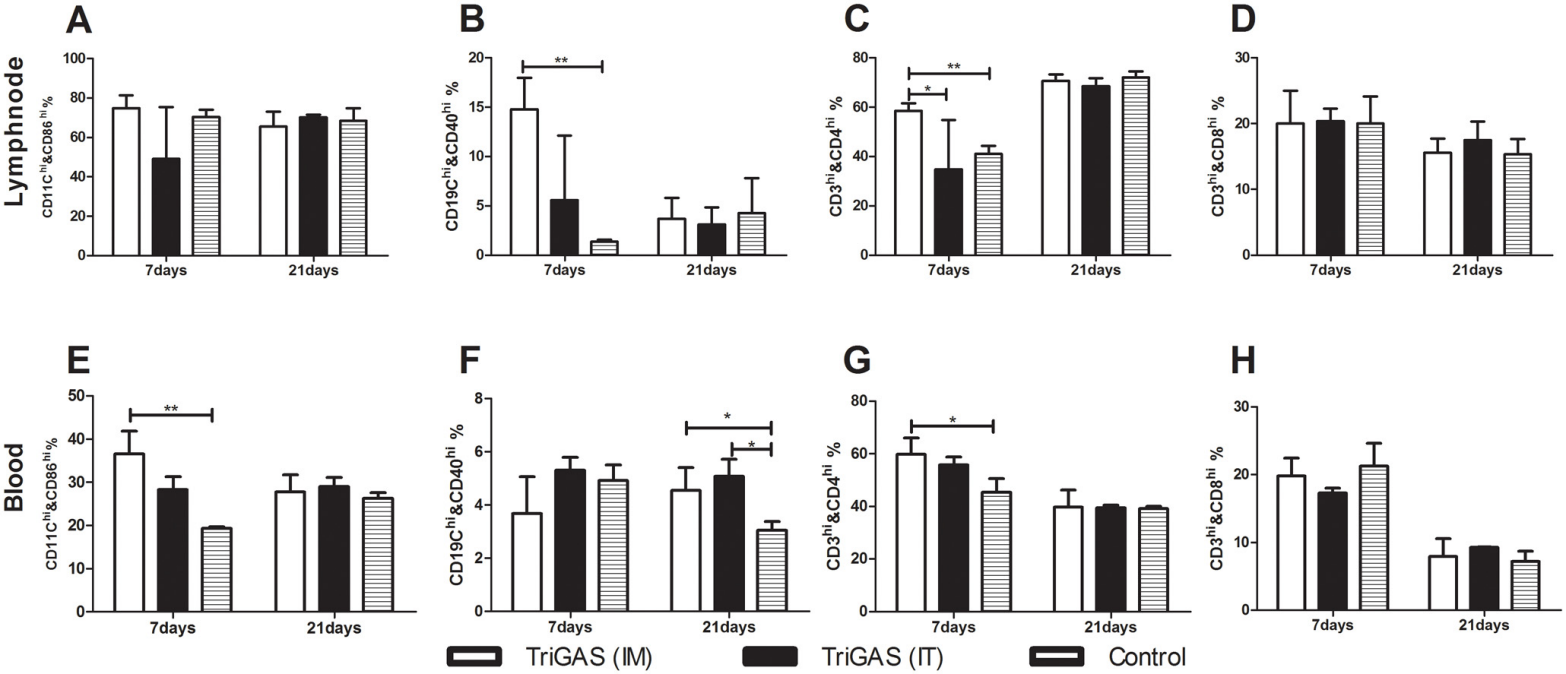
Comparison of cell-mediated immune responses to TriGAS by IM and IT route of immunization. Groups of dog were vaccinated with TriGAS via IM or IT route and compared the activation status of DCs and B-cells, and number of CD4 and CD8 T-cells in the lymph node (A-D) and in the blood (E-H).

In order to compare the activation status of immune cells to TriGAS by IM and IT immunization, blood and LN samples were used for flow cytometric analysis. As shown in Fig 5, significantly higher numbers of activated B cells and DCs, and CD4 T cells in the lymph node and the blood were detected in IM immunized dogs than in the uninfected control or in the IT immunized dogs at 7 days post vaccination (Fig 5B, 5C, 5E and 5G). However, a significantly greater number of activated B cells was detected in IM and IT immunized dogs than in the uninfected controls at 21 days post vaccination (Fig 5F). These results indicate that the IT vaccination induces activation of B cells in the peripheral blood at later stages of infection.

## Discussion

Rabies is a highly lethal but preventable disease caused by the neurotropic RABV [38, 39]. RABV infects the CNS, causing fatal encephalitis and death [40]. However, the mechanism of rabies pathogenesis is still not completely understood. For the past several years, remarkable advances have been made in elucidating the pathogenesis of rabies. Animals infected with wt-RABVs do not develop VNA, whereas attenuated RABVs induce high levels of VNA. In this present study, we compared the immune responses in dogs after infection with an attenuated TriGAS that is non-pathogenic in nature or a wt DRV-NG11 which is a highly pathogenic virus isolated from a rabid dog [31]. Consistent with a previous report in mice [41], our study shows IM infection of TriGAS in dogs induced a high level of serum VNA. However, wt RABV infection in dogs did not induce any VNA production in the serum or CSF. Similarly, the inability of wt RABV to induce VNA responses has also been reported in other animal species such as mice [11] and skunks [9]. Also, it is well known that rabies patients rarely develop rabies VNA [42]. Furthermore, TriGAS infection in dogs induced antibodies against RABV including its G protein, whereas the DRV-NG11 infection induced anti-RABV antibodies but was neither significantly different from the TriGAS infected dogs nor from the uninfected controls. However, DRV-NG11 did not induce any antibodies against rabies G protein.

Previous studies including those from the Hooper’s laboratory have shown that Lyssaviruses evade the host immune response by maintaining the integrity of BBB in the mouse model [43]. However, BBB permeability was measured on 8 dpi or at earlier time points in mice before developing diseases. In the present studies, dogs were infected with wt RABV and BBB integrity was monitored at 7 dpi and at the time of termination (severe disease). BBB integrity was normal at 7 dpi but was severely damaged at the terminal stage of disease. The discrepancies reported between these studies could be due to a number of factors. The animals used in previous studies are mice and those in the present study are dogs. Wt RABV may induce different damages to the BBB integrity in different animal species. Alternatively, BBB was measured in mice prior to the development of diseases and BBB damage has not occurred at that time. Further studies may be needed to address these issues. Nevertheless, dogs infected with wt RABV did not develop VNA even at the terminal stage of infection and no VNA was detected in either the serum or the CSF. It has been reported that enhancement of BBB permeability and the presence of VNA are required to clear RABV from the CNS [11, 44]. Thus enhancement of BBB permeability alone without the presence of VNA in these dogs is not sufficient to protect these animals from developing rabies.

Although the mechanism(s) by which different RABV triggers and evades immune responses are unknown, studies [45–47] indicate that laboratory-attenuated RABVs are capable of triggering host innate immune responses, while wt RABV evades immune responses [46, 47]. It has been shown using recombinant RABVs expressing chemokines that promote activation of immune cells, especially DCs & B cells, correlates with higher level of VNA production and better protection [13, 48, 49].

Next, we examined the cellular immune response to attenuated or wt RABV infection, particularly the activation status of DCs and B cells, and the number of CD4 and CD8 T cells in blood and lymph nodes. TriGAS infection in dogs induces early activation of B cells in the lymph nodes and persistent activation of DCs in the blood. Similar observations have been observed in mice infected with TriGAS and other attenuated recombinant RABVs [13, 48, 49]. It has been shown in mice that IM inoculation of TriGAS resulted in transient infection of stroma, B cells and DCs in the lymph nodes as detected by the quantification of viral mRNA and genomic RNA [41]. Based on the current observation on B cell activation in the draining lymph nodes, DC maturation in the peripheral blood, and VNA production in the serum, it is plausible that similar processes are likely to take place during immunization with TriGAS in dogs. Thus TriGAS infection induces DC activation, thereby resulting in B cell activation and VNA production. In addition, CD4 T cells have been shown to play a crucial role in the development of protective immunity against rabies [50, 51]. Indeed, a significant increase in the number of CD4 T cells was observed in TriGAS-infected dogs. These results indicate that the attenuated RABVs stimulate the innate/adaptive immune responses.

On the other hand, wt RABV (DRV-NG11) infection did not induce activation of immune cells, rather it reduced the number of activated DCs, B cells, and CD4 T cells. It is evident from the lack of antibodies induced after rabies infection in most humans [42] or in laboratory animals [7, 9, 11] that the wt RABVs can efficiently evade the host immune responses. Although a number of factors could limit the responsiveness to infection, many studies indicate the role of immune suppression by RABVs. Earlier studies in the late 1980s have shown RABV mediated lymphoid depletion [52–54]. It has been shown that the depletion of lymphocytes or the depression of cell-mediated immunity in mice during wt RABV infection is associated with lymphocyte apoptosis [55]. Also, it has recently been shown that the RABV phosphoprotein can interact with STAT1 and inhibit the interferon signaling pathways [56, 57]. It has been shown in the brain and spinal cord, that the rabies infection can significantly up-regulates the expression of immune-inhibitory molecule B7-H1 [58]. Particularly, our results show that the suppression of immune cell activation occurs only at the later point of infection. We speculate that wt RABVs which expresses limited amount of its G protein than the attenuated RABVs could lead to poor presentation of its antigens to T cells, which could further lead to suboptimal stimulation. Further studies are warranted to define the detailed mechanisms of wt RABV-mediated immune evasion.

Although rabies is traditionally considered a uniformly fatal disease after onset of clinical manifestation [59, 60], non-lethal human rabies cases in humans [22–24] and laboratory animals such as mice [25], ferrets [26], and dogs [27, 28] have been documented. However, all these cases had rabies-specific VNA in the CSF. Thus, the induction of CSF VNA seems to be one of the crucial factors for RABV clearance from CNS and recovery from rabies. In this study, the comparison of the immune responses elicited by IM and IT immunization of TriGAS in dogs shows adequate level of VNA in the serum but not in the CSF in IM-vaccinated dogs, however, IT vaccination induced adequate level of VNA both in the serum and CSF. In addition, IT vaccination not only induced VNA production in the CSF but also enhanced the BBB permeability, as observed by the presence of total proteins and WBCs in the CSF. Similar IT vaccination in dogs has been reported by Baer et al., which not only induced VNA in the CSF, but also prolonged the morbidity [61]. Recent report of survival of rabid rabbits after IT immunization highlights the importance of CSF VNA on viral clearance [30]. Furthermore, it has recently been demonstrated that the RABV infection can be cleared from the CNS by exogenous administration of VNA in immunocompetent or immunocompromised mice, as long as the BBB permeability is enhanced for the passage of VNA from the periphery to the CNS [29]. Thus, the induction of CSF VNA via IT vaccination could be an important step towards developing future therapeutics for the clearance of RABV from the CNS.
